# Reconstructing the phylodynamic history and geographic spread of the CRF01_AE-predominant HIV-1 epidemic in the Philippines from PR/RT sequences sampled from 2008 to 2018

**DOI:** 10.1093/ve/vead073

**Published:** 2023-12-07

**Authors:** Francisco Gerardo M Polotan, Carl Raymund P Salazar, Hannah Leah E Morito, Miguel Francisco B Abulencia, Roslind Anne R Pantoni, Edelwisa S Mercado, Stéphane Hué, Rossana A Ditangco

**Affiliations:** Molecular Biology Laboratory, Research Institute for Tropical Medicine, 9002, Research Drive, Filinvest Corporate City, Alabang, Muntinlupa City, Metro Manila 1781, The Philippines; Laboratory of Microbiology, Wageningen University and Research, Stippeneng 4, Wageningen 6700 EH, The Netherlands; Molecular Biology Laboratory, Research Institute for Tropical Medicine, 9002, Research Drive, Filinvest Corporate City, Alabang, Muntinlupa City, Metro Manila 1781, The Philippines; Molecular Biology Laboratory, Research Institute for Tropical Medicine, 9002, Research Drive, Filinvest Corporate City, Alabang, Muntinlupa City, Metro Manila 1781, The Philippines; Molecular Biology Laboratory, Research Institute for Tropical Medicine, 9002, Research Drive, Filinvest Corporate City, Alabang, Muntinlupa City, Metro Manila 1781, The Philippines; Molecular Biology Laboratory, Research Institute for Tropical Medicine, 9002, Research Drive, Filinvest Corporate City, Alabang, Muntinlupa City, Metro Manila 1781, The Philippines; Centre for the Mathematical Modelling of Infectious Diseases (CMMID), London School of Hygiene & Tropical Medicine, Keppel Street, London, Camden WC1E 7HT , UK; Department of Infectious Disease Epidemiology, London School of Hygiene & Tropical Medicine, Keppel Street, London, Camden WC1E 7HT , UK; AIDS Research Group, Research Institute for Tropical Medicine, 9002, Research Drive, Filinvest Corporate City, Alabang, Muntinlupa City, Metro Manila 1781, The Philippines

**Keywords:** HIV-1, epidemic, CRF01_AE, Subtype B, phylodynamics, molecular epidemiology, Philippines

## Abstract

The Philippines has had a rapidly growing human immunodeficiency virus (HIV) epidemic with a shift in the prevalent subtype from B to CRF01_AE. However, the phylodynamic history of CRF01_AE in the Philippines has yet to be reconstructed. We conducted a descriptive retrospective study reconstructing the history of HIV-1 CRF01_AE transmissions in the Philippines through molecular epidemiology. Partial polymerase sequences (*n *= 1144) collected between 2008 and 2018 from three island groups were collated from the Research Institute for Tropical Medicine drug resistance genotyping database. Estimation of the time to the most recent common ancestor (tMRCA), effective reproductive number (*R*_e_), effective viral population size (*N*_e_), relative migration rates, and geographic spread of CRF01_AE was performed with BEAST. *R*_e_ and *N*_e_ were compared between CRF01_AE and B. Most CRF01_AE sequences formed a single clade with a tMRCA of June 1996 [95 per cent highest posterior density (HPD): December 1991, October 1999]. An increasing CRF01_AE *N*_e_ was observed from the tMRCA to 2013. The CRF01_AE *R*_e_ reached peaks of 2.46 [95 per cent HPD: 1.76, 3.27] in 2007 and 2.52 [95 per cent HPD: 1.83, 3.34] in 2015. A decrease of CRF01_AE *R*_e_ occurred in the intervening years of 2007 to 2011, reaching as low as 1.43 [95 per cent HPD: 1.06, 1.90] in 2011, followed by a rebound. The CRF01_AE epidemic most likely started in Luzon and then spread to the other island groups of the country. Both CRF01_AE and Subtype B exhibited similar patterns of *R*_e_ fluctuation over time. These results characterize the subtype-specific phylodynamic history of the largest CRF01_AE cluster in the Philippines, which contextualizes and may inform past, present, and future public health measures toward controlling the HIV epidemic in the Philippines.

## Introduction

Human immunodeficiency virus-1 (HIV-1) infections and deaths related to acquired immunodeficiency syndrome (AIDS) have been rapidly increasing in the Philippines over the past years, with a 237 per cent percent change in new infections, the highest in Asia and the Pacific from 2010 to 2020 (Epidemiology Bureau DoH 2020a). As of June 2022, 101,768 confirmed HIV cases have been reported since January 1984, with about 92 per cent of these reported in the last 10 years and the number of new diagnoses increasing from six cases/day in 2011 to forty-one cases/day in 2022 (Epidemiology Bureau DoH 2022).

The demographics of HIV-1 cases in the Philippines have also shifted over time. From 1984 to 1990, the majority of diagnosed cases (62 per cent) were females, compared to a majority of male cases (94 per cent) in 1991–2020 (Epidemiology Bureau DoH 2020b). The largest proportion of new cases also shifted to a younger age group, from 35–49 in 2001–5 to 25–34 and 15–24 age groups in 2010–22 (Farr and Wilson 2010; Ross et al. 2013, 2015; Epidemiology Bureau DoH 2022). Furthermore, the majority of transmissions were from male-to-female sex until 2007 (Ross et al. 2013; Epidemiology Bureau DoH 2022), with men who have sex with men (MSM) continuing to grow in proportion as the largest mode of transmission in the Philippines from that time onward (Epidemiology Bureau DoH 2020b).

The composition of circulating subtypes in the Philippines has also changed. In 1998, the major subtype was B, with around 70 per cent of infections, followed by CRF01_AE, a putative recombinant of Subtypes A and E, with 16–29 per cent of infections (Paladin et al. 1998; Santiago et al. 1998). However, the major subtype has shifted to CRF01_AE, making up 77 per cent of HIV-1 patients in a 2013 cohort and with 22 per cent of the same cohort being Subtype B (Salvaña et al. 2017). A more recent cohort from 2016 to 2018 reported the distribution of 77 per cent CRF01_AE, 13.8 per cent Subtype B, and 9.2 per cent other subtypes or recombinants (Salvaña et al. 2022). Furthermore, CRF01_AE was the major subtype among the MSM population, while B was predominant among persons who inject drugs in infections from 2007 to 2012 (Telan et al. 2013; Salvaña et al. 2017). In the Philippines, CRF01_AE has been found to be significantly associated with higher viral load (>100,000 copies/ml) and lower CD4 count (<50 cells/mm^3^) compared to other circulating subtypes (Salvaña et al. 2022). There is also a high rate of tenofovir (TDF) treatment failure (Odds Ratio: 3.28, 95 per cent Confidence Interval: 1.58–7.52, *P* < 0.001) and higher rates of K65R and multiclass drug resistance mutations in the Philippine CRF01_AE-predominant epidemic compared to failure rates reported by a WHO systematic review (about 40 per cent to 7 per cent and about 80 per cent to 49 per cent, respectively) (World Health Organization 2017; Salvana et al. 2020; Salvaña et al. 2022). Additionally, CRF01_AE in the Philippines was suggested to have a trend of high transmitted drug resistance (TDR) compared to Subtype B (Salvaña et al. 2022). With WHO recommendations based on Subtype B infections and TDF as a universal first-line antiretroviral (ARV) treatment (World Health Organization 2016), researchers have called for further investigation of the potential subtype-specific impact of current ARV regimens in light of these trends (Salvaña et al. 2017, 2020).

Based on previous studies, CRF01_AE may have been introduced into the Philippines around the mid-1990s, potentially from a neighboring Asian or Southeast Asian country (Chen et al. 2019; Junqueira et al. 2020). The Chen et al. (2019)’s study estimated the CRF01_AE time to the most recent common ancestor (tMRCA) using fourteen nearly full-length genome sequences of CRF01_AE sampled over 3 years (2015–7) from Luzon, while the Junqueira et al. (2020)’s study used four near complete genome sequences from the Philippines sampled from 2015 to 2016 without a specific island group indicated. Although whole-genome sequences would be able to more accurately distinguish recombinant sequences (Topcu et al. 2022) and lead to more precise estimates (Dudas and Bedford 2019), partial *pol* sequences from drug resistance genotyping (DRG) have been shown sufficient to reconstruct transmission histories phylogenetically and are highly available data from routine testing (Hué et al. 2004). The Research Institute for Tropical Medicine (RITM), one of the largest referral hubs in the Philippines, has archived over a thousand *pol* protease/reverse transcriptase (PR/RT) sequences in its DRG database from routine testing of patient samples referred to the RITM between 2008 and 2018 across all three Philippine island groups, compiling a suitable and available dataset for reconstructing subtype-specific phylodynamics. Despite the predominance of CRF01_AE among the circulating subtypes in the Philippines, to the best of our knowledge, there has not yet been any study disentangling epidemiological parameters of specific HIV subtypes in the Philippines using phylodynamic methods.

Thus, we aimed to estimate the CRF01_AE tMRCA as well as reconstruct changes in its effective viral population size (*N*_e_), effective reproductive number (*R*_e_), relative migration rates, and phylogeographic spread using over a thousand CRF01_AE partial *pol* sequences sampled over a decade, including sequences from all three Philippine island groups. We also compared these with the *N*_e_ and *R*_e_ of Subtype B to compare and contrast the dynamics of each HIV subtype and provide important context to public health policies attempting to control its spread in the country.

## Methods

### Study population and sample selection

The retrospective study population was HIV-positive cases from the in-house RITM HIV-1 DRG database with 1530 cases matching the inclusion criteria (see the following section) for CRF01_AE analysis, while 265 cases matched the inclusion criteria for the comparative Subtype B analysis. The data included PR/RT Sanger sequences generated from RITM routine DRG with accompanying information on specimen collection date. Collated data for CRF01_AE analysis consisted of sequences from patient samples from RITM and from referring hospitals and social hygiene clinics from thirteen out of seventeen administrative regions from all three Philippine island groups between January 2008 and November 2018. Meanwhile, data for the Subtype B analysis came from RITM patients and patients from referring hospitals and social hygiene clinics from ten out of seventeen administrative regions from all three Philippine island groups between February 2014 and February 2020.

Demographic data (i.e. sample collection date and requisitioner address as location) were obtained via request forms. Missing demographic data from the samples were requested from the Department of Health Epidemiology Bureau. People living with HIV have unique HIV laboratory test ID numbers, and these were used to obtain data from the Epidemiology Bureau without using patient names.

The institutional review board of the RITM waived ethical approval for this study by granting the study protocol a certificate of exemption from review as it was a retrospective analysis of archived de-identified routine DRG sequence data and metadata only.

### Subtype classification and sequence alignment

The inclusion criteria used were as follows: all PR/RT *pol* sequences available in the RITM Molecular Biology Laboratory (MBL) DRG; sequences from RITM classified as CRF01_AE or Subtype B; sequences at least 500 nucleotides long. The exclusion criteria were as follows: non-CRF01_AE or Non-Subtype B sequences from RITM MBL DRG, sequences less than 500 nucleotides long; Los Alamos National Laboratory (LANL) HIV Sequence Database (Foley et al. 2018) sequences without country and year of sampling information; sequences containing ≥5 per cent ambiguous nucleotides, frameshift mutations, stop codons, and APOBEC-mediated hypermutations. The remaining sequences that passed these filters all span the PR/RT sequences, specifically Nucleotide Coordinates 2253 to 3233 or PR Codon 1 to RT Codon 227 of sequence HXB2 (K03455.1) (Ratner et al. 1985, 1987; Kuiken, Korber, and Shafer 2003).

Thus, partial *pol* sequences from the RITM DRG database (*n *= 1621) were classified according to HIV subtype (i.e. phylogenetically related strains defined by close genetic distance with each other) using the Stanford HIV Drug Resistance Database (Rhee et al. 2003) and HIVdb program (v8.6) (Liu and Shafer 2006), and the programs COMET (Struck et al. 2014) and REGA (Pineda-Peña et al. 2013) were used to exclude non-CRF01_AE and recombinant sequences, respectively. The ten most closely related CRF01_AE *pol* gene sequences (HXB2 Coordinates 2253 to 3233) per input sequence were also retrieved from the LANL HIV Sequence Database (Foley et al. 2018) in January 2019 using HIV-BLAST. LANL sequences (*n *= 454) from 1990 to 2016 with information on the sampling year and country and without >5 per cent ambiguous nucleotides, frameshift mutations, stop codons, and APOBEC-mediated hypermutations were retrieved. Sequences that contained frameshift insertions/deletions as determined by HIVdb were excluded. A small number of sequences that had very few bases in the PR/RT region or had very long insertions were also excluded. A codon alignment of all remaining sequences was produced with the Gene Cutter tool from LANL (Los Alamos National Laboratory 2019). A 129 bp-long stretch of sequence, spanning reference HXB2 Codon Position 96 of the PR protein sequence to Codon Position 39 of the RT protein sequence (HXB2 Nucleotide Coordinates 2538 to 2666), was removed from the alignment since a majority of the Philippine sequences in this study contained gaps in this region. This was due to the nested reverse transcription polymerase chain reaction protocol that was used to generate these sequences (Steegen et al. 2006). Drug resistance–associated codons were stripped to remove the influence of convergent evolution from drug resistance mutations (Wensing et al. 2016). Large overhangs were trimmed using Aliview (Larsson 2014), and the sequences from the same patient were removed, resulting in 1429 sequences.

Using similar procedures, high-confidence Subtype B sequences were identified (*n *= 265) for the comparative analysis. The ten most similar Subtype B *pol* sequences per input sequence were also retrieved from LANL in May 2021 using HIV-BLAST (*n *= 599). Codon alignment, filtering, and trimming were also performed. Five partial polymerase sequences each from Subtype A (DQ396400, DQ445119, JQ403028, KX389622, and MH705133) and Subtype C (AB023804, AB097871, AB254143, AB254146, and AB254150) were included as outgroups for subsequent phylogenetic tree reconstruction, leading to 874 sequences. Out of these 874 sequences, 316 were sampled from the Philippines either by this study or retrieved from LANL, 548 were international Subtype B sequences, and 10 were outgroup sequences.

For both CRF01_AE and Subtype B sequences, collection dates with complete year and month but not day were imputed to the 15th day of the month, while collection dates with complete year only but not month and day were imputed to 15th of June of the year.

### Phylodynamic analysis

IQ-TREE 2 (Minh et al. 2020) was used to reconstruct a maximum likelihood phylogeny from the full CRF01_AE alignment containing Philippine and international sequences. The full set of Philippine CRF01_AE sequences from the largest monophyletic clade that have sample collection dates (*n *= 1144) was used to estimate the time to the most recent common ancestor (tMRCA) of that clade as well as the effective viral population size (*N_e_*). TempEst v1.5 (Rambaut et al. 2016) was used to measure the temporal signal of these sequences by root-to-tip regression analysis. BEAST v2.6.7 (Bouckaert et al. 2014) was used to estimate the tMRCA and *N*_e_, with a coalescent Bayesian skyline plot (BSP) tree prior (Drummond et al. 2005), bModelTest (Bouckaert and Drummond 2017) set as the nucleotide substitution model, clock rate prior set to a normal distribution with *M* = 1.5E − 3, *S* = 4.9E − 4 following previous estimates of the evolutionary rate of HIV-1 *pol* gene sequences (Jenkins et al. 2002; Dalai et al. 2009; Jung et al. 2012; Patiño-Galindo and González-Candelas 2017), clock rate standard deviation prior to an exponential distribution with *M* = 0.1 following the prior set in ), and the best-fitting molecular clock model of uncorrelated log normal distribution relaxed molecular clock (ucld) (Drummond et al. 2006). The best-fitting clock model was determined by the path sampling and stepping-stone procedure (Lartillot, Philippe, and Lewis 2006; Xie et al. 2011) implemented in BEAST v1.10.4 (Suchard et al. 2018). The bPopSizes and bGroupSizes were both left at the dimension of 5. Twenty-four separate Markov chain Monte Carlo (MCMC) chains were run for 300 m states each, sampling every 30,000 states. These were combined with 20 per cent burnin each and then downsampled to 8,000 states and trees using LogCombiner (Bouckaert et al. 2014). TreeAnnotator (Bouckaert et al. 2019) was used to generate the maximum clade credibility (MCC) tree. The resulting BSP *N*_e_ estimate was plotted up to the last coalescent event in the CRF01_AE MCC tree, which was in mid-2017.

To estimate the effective reproductive number (*R*_e_) of CRF01_AE across time, the same set of 1144 Philippine sequences belonging to the large Philippine monophyletic clade was used. BEAST2 was run with the birth-death skyline (BDSKY) serial (Stadler et al. 2013) tree prior, bModelTest (Bouckaert and Drummond 2017) selected as the nucleotide substitution model, the best-fitting molecular clock model set to the uncorrelated log normal distribution relaxed molecular clock (Drummond et al. 2006), and the clock rate prior set to as indicated previously. The origin of the epidemic prior was set to a log normal distribution with *M* = 3.4, *S* = 0.29, centering the distribution to 30 years before the latest sampled date, which is slightly earlier than the upper bound of the tMRCA from a previous study (Junqueira et al. 2020). The becoming uninfectious rate prior was set to a log normal distribution in real space with *M* = 2.08, *S* = 1.0, covering a range of values estimated for HIV-1 from previous studies (Stadler et al. 2013; Wilkinson et al. 2019; Vasylyeva et al. 2020). The reproductive number prior was set to a log normal distribution with *M* = 0.0 in log space, *S* = 1.25 to center the *R*_e_ prior at 1.0 for a null hypothesis of a constant epidemic size (Barido-Sottani et al. 2018). The number of reproductive number dimensions was left at 10. The TreeSlicer utility of the skylinetools package (du Plessis 2018) was used to set three sampling proportion intervals. The first sampling interval was from the origin to the first sample collected in 15 January 2008. The second and third sampling intervals were equidistant intervals from the first sample collected to the date 17 June 2013 and from the date 17 June 2013 to the most recent sample collected in 18 November 2018, respectively. In the first sampling interval with no sampling, sampling proportion was set to zero, while in the second and third sampling intervals, the sampling proportion prior was set to a beta distribution with alpha = 1.0, beta = 99.0. This distribution includes values from below 0.02 per cent (2.5 per cent quantile) to above 3.66 per cent (97.5 per cent quantile), with a mean of 1 per cent and biased toward 0 per cent, since the sequence datasets used in the study are limited to only hundreds to over one thousand sequences compared to the cumulative HIV-1 infections projected to be over one hundred thousand in the Philippines (Epidemiology Bureau DoH 2020a). Nineteen separate MCMC chains were run for 300 m states each, sampling every 30,000 states. These were combined with 20 per cent burnin each and no downsampling using LogCombiner (Bouckaert et al. 2019). The MCC tree was generated from sampled trees using TreeAnnotator (Bouckaert et al. 2019). The bdskyTools R package was used to smooth the *R*_e_ estimates over time (Du Plessis 2018).

Two spatiotemporally uniform subsamples of CRF01_AE sequences were also used in BSP and BDSKY analyses to assess the robustness of *N*_e_ and *R*_e_ trends observed from the full dataset. The sequences from the large monophyletic clade were subsampled uniformly across time and geographic location (Hall, Woolhouse, and Rambaut 2016; Hidano and Gates 2019) according to the island group (i.e. Luzon, Visayas, and Mindanao) and between May 2008 and November 2018. Specifically, the uniform subsampling procedure outlined by Hidano and Gates (2019) was used, producing a subset of 256 sequences. A second uniform subsample was generated by excluding the sequences in the aforementioned subsample and repeating the uniform sampling procedure on the remaining sequences, producing a subset of 151 sequences. The two subsamples are non-overlapping sets except for two sequences that have the earliest and latest sample collection dates across the whole dataset (15 January 2008 and 18 November 2018). These were included in both subsamples in order to set the same changepoint times and sampling proportion slices as the full dataset with TreeSlicer (du Plessis 2018). The same models, priors, MCMC chain length, and MCMC sampling frequency as in the full dataset were selected. Four and five independent MCMC chains were combined with 10 per cent burnin each and then downsampled to 9,000 states and trees using LogCombiner (Bouckaert et al. 2019) for the BSP and BDSKY analysis of the 256 sequence subsample, respectively. One and three independent MCMC chains were run with 10 per cent burnin each sampling 9,000 states and trees for the BSP and BDSKY analysis of the 151 sequence subsample, respectively.

To compare the CRF01_AE *N*_e_ and *R*_e_ with those of another circulating subtype that may reflect similar patterns if non-subtype-specific influences were acting upon their transmission, an equivalent analysis was done using BEAST2 on the largest Philippine Subtype B clade identified from a phylogeny reconstructed using IQ-TREE 2. Out of the 197 Philippine Subtype B sequences under this clade, two had no sample collection dates and were excluded. Three outlier sequences identified using TempEST root-to-tip regression were also excluded. This led to only 192 Philippine Subtype B sequences remaining in this clade, and thus no subsampling was performed. The same respective models, priors, MCMC chain length, and MCMC sampling frequency as in the BSP and BDSKY CRF01_AE analyses were used. The best-fitting clock model for the Subtype B dataset was also determined to be the uncorrelated log normal distribution relaxed molecular clock by the path sampling and stepping-stone procedure (Lartillot, Philippe, and Lewis 2006; Xie et al. 2011). For the BDSKY analysis, TreeSlicer (du Plessis 2018) was used to set three sampling proportion intervals: the first sampling interval from the origin to the first sample collected in 29 July 2008, the second sampling interval from 29 July 2008 to the date of 02 May 2014, and the third sampling interval, an equidistant interval, from 02 May 2014 to the most recent sample collected in 03 February 2020. As in the CRF01_AE dataset, the first sampling interval was set to zero, while the second and third intervals were estimated in BDSKY. One MCMC chain was run with 10 per cent burnin for both BSP and BDSKY analyses. The resulting BSP *N*_e_ estimate was plotted up to the last coalescent event in the Subtype B MCC tree, which was in 2018. The MCC tree was generated using TreeAnnotator (Bouckaert et al. 2019), and bdskyTools was used to plot *R*_e_ across time (Du Plessis 2018).

### Phylogeographic analysis

We tested for significant associations (α = 0.05) of island group and tree topology using the Bayesian tip-association significance testing (Befi-BaTS) package (Parker, Rambaut, and Pybus 2008), which calculated the association index (AI), parsimony score (PS), and monophyletic clade (MC) index, unique fraction (UniFrac), nearest taxa (NT) index, net relatedness (NR) index, and phylogenetic diversity (PD) indices on 1,000 downsampled trees from the BSP analyses of the full 1144 CRF01_AE sequence dataset and the 256 uniform subsample of CRF01_AE sequences using 999 null replicates. The few sequences with no location metadata in these datasets were assigned to ‘NA’.

BEAST v1.10.4 (Suchard et al. 2018) was used for the phylogeographic analysis of the full CRF01_AE dataset alignment with nucleotide positions and demes set as the first and second partitions, respectively. Of the 1144 CRF01_AE sequences from the large Philippine clade with sample collection dates, only 1042 also had island group metadata and were included in the phylogeographic analysis (Luzon = 933, Visayas = 34, Mindanao = 75). Analysis was performed using the uncorrelated log normal distribution relaxed molecular clock model and a BSP tree prior. The asymmetric discrete trait substitution model with Bayesian Stochastic Search Variable Selection was selected for using Philippine island groups as demes. The population size dimension was set to 3. Thirty separate MCMC chains were run for 300 m states each, sampling every 30,000 states. These were combined with 40 per cent burnin each and then downsampled with LogCombiner (Suchard et al. 2018) to 6,000 states and trees. To assess the robustness of the phylogeographic inferences, a sensitivity analysis on ten different subsamples were conducted, where a balanced number of sequences (*n* = 34) were selected randomly from each island group for a total of 102 sequences per subsample. The same analysis was also performed on the first uniform subsample of CRF01_AE sequences but excluding the earliest sampled sequence from 15 January 2008 since it did not have location metadata (*n* = 255). Phylogeographic analysis was performed on each subsample with the same models, priors, MCMC chain length, and MCMC sampling frequency as in the full dataset. Three and one MCMC chain was run with 10 per cent burnin each for the uniform subsample of 255 sequences and the ten balanced subsamples of 102 sequences, respectively. TreeAnnotator (Suchard et al. 2018) was used to generate the MCC tree for each dataset. SPREAD4 (Nahata et al. 2022) was used to visualize phylogeographic migration across three island groups over a map of the Philippines ().

Other settings across all analyses besides those stated were left at default values. An effective sample size of ≥200 was deemed as satisfactory for the convergence of the estimated parameters. The Tracer (Rambaut et al. 2018), FigTree (Rambaut 2010), and IcyTree (Vaughan and Valencia 2017) software and R packages ‘ggtree’ (Yu et al. 2017) and ‘ggplot2’ (Wickham 2016) were used to visualize the results.

## Results

### The introduction and growth of the largest CRF01_AE cluster in the Philippines

**Figure 1.**
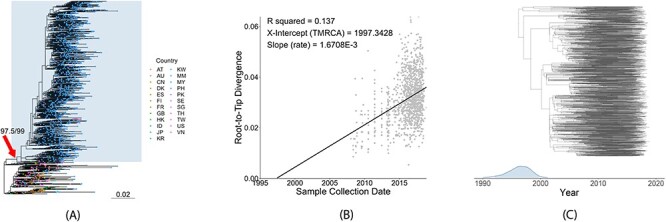
(A) The maximum likelihood phylogenetic tree of Philippine CRF01_AE PR/RT sequences relative to international sequences obtained from the LANL database reconstructed with IQ-TREE 2. The arrow indicates the node, with SH-aLRT and UFBoot branch support values, of the most recent common ancestor of the large MC to which the majority of CRF01_AE sequences belong. This clade is also emphasized with a rectangular highlight. The bottom scale bar shows a reference branch length for 0.02 substitutions/site. Country abbreviations: AT: Austria; AU: Australia; CM: Cameroon; CN: China; DK: Denmark; ES: Spain; FI: Finland; FR: France; GB: United Kingdom; HK: Hong Kong; ID: Indonesia; JP: Japan; KR: South Korea; KW: Kuwait; MM: Myanmar; MY: Malaysia; PH: Philippines; PK: Pakistan; SE: Sweden; SG: Singapore; TH: Thailand; TW: Taiwan; US: USA; VN: Vietnam. (B) Root-to-tip plot generated with TempEst showing a positive correlation between time and divergence or accumulating mutations among sequences, indicating suitability of sequence data for time-scaled phylogenetic and phylodynamic analysis. (C) Posterior probability estimate of the tMRCA of the large Philippine CRF01_AE clade with corresponding MCC tree generated using BEAST2 from the full dataset of 1144 Philippine CRF01_AE sequences.

**Figure 2.**
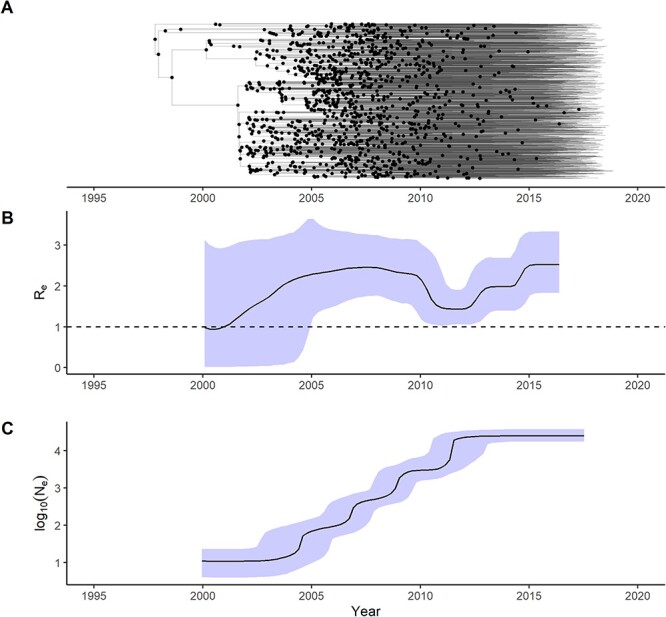
Phylodynamics of the largest HIV CRF01_AE cluster in the Philippines between the mid-to-late 1990s and 2018 reconstructed by BEAST2 analysis of the full 1144 sequence dataset of Philippine CRF01_AE PR/RT sequences. (A) Time-scaled MCC tree from BDSKY analysis summarized with TreeAnnotator. Common ancestor internal nodes are highlighted as dots. (B) The change in the effective reproductive number (*R*_e_) over time, with the median represented as a solid line and the 95 per cent HPD as a shaded interval, obtained from BDSKY analysis. A dashed line indicates the value of *R*_e_ equal to 1. (C) The change in CRF01_AE effective population size (*N*_e_) over time in log scale, with the median represented as a solid line and the 95 per cent HPD as a shaded interval, obtained from BSP analysis.

Out of all the available Philippine CRF01_AE sequences, the majority (1144/1179; 97 per cent) belonged to one large MC (Fig. 1A; SH-aLRT, UFBoot branch support: 97.5, 99). Fewer sequences (35/1179; 3 per cent) were singletons or belonged to smaller clusters of at most three sequences, suggesting multiple introductions from overseas over time. Out of all sequences in this large clade, the majority were Philippine sequences (1144/1183, 96.7 per cent), while a smaller proportion were international sequences (39/1183, 3.3 per cent) from the USA, Japan, Hong Kong, Australia, Great Britain, China, and South Korea, suggesting transmission to these countries (Fig. 1A). Root-to-tip linear regression on the full set of Philippine sequences belonging to the large clade showed a positive correlation between sequence diversity and time of sampling (coefficient of correlation: 0.37; *R*^2^ = 0.137), indicating sufficient temporal signal for further molecular clock estimations (Fig. 1B). Coalescent BSP analysis was conducted on the large Philippine cluster to estimate the tMRCA and *N*_e_. The tMRCA was estimated to have a median of June 1996 [95 per cent highest posterior density (HPD): December 1991, October 1999] (Fig. 1c, Supplementary Table S1), and the estimated evolutionary rate had a median of 3.2664E − 3 [95 per cent HPD: 2.9275E − 3, 3.5935E − 3] nucleotide substitutions/site/year (Supplementary Table S1). The resulting skyline plot, showing changes of effective population size over time, revealed a growth phase with an increasing *N*_e_ from the tMRCA to about 2014 (Fig. 2C). From 2014 to 2017, a plateau in CRF01_AE *N*_e_ is observed (Fig. 2C).

### Fluctuations of CRF01_AE *R*_e_ from the 1990s to 2015

Phylodynamic analysis was performed with the same full dataset of 1144 CRF01_AE sequences from the large Philippine clade using the BDSKY model. The *R*_e_ of CRF01_AE increased from 1.0 around the time of the tMRCA to about 2.46 [95 per cent HPD: 1.76, 3.27] in 2007, indicating a rapid growth in the largest CRF01_AE cluster during this period (Fig. 2B). Following this peak in 2007, the *R_e_* decreased to as low as 1.43 [95 per cent HPD: 1.06, 1.90] in 2011 (Fig. 2B), although this estimate remained above an *R*_e_ of 1.0. Finally, from 2012 to 2015, the *R*_e_ rebounded reaching a second peak of 2.52 [95 per cent HPD: 1.83, 3.34] in 2015 (Fig. 2B). In order to assess the robustness of these observed *R*_e_ trends, BDSKY analysis using the same models and prior parameters was also performed for two distinct spatiotemporally uniform subsamples, both showing the same general trend of an increasing, decreasing, and rebounding *R*_e_ as in the full dataset (Supplementary Fig. S2).

### Migration of CRF01_AE from Luzon to Visayas and Mindanao

Using the Befi-BaTS test on the full dataset of 1144 CRF01_AE sequences, significant clustering of sequences by island group was observed for the AI, PS, NT, PD, MC Luzon, MC Visayas, and MC Mindanao indices, while the UniFrac and NR indices did not reach statistical significance (Supplementary Table S2). Similarly, significant clustering by island group was also observed in the uniform subsample of 256 CRF01_AE sequences for the AI, PS, NT, NR, PD, MC Luzon, and MCMindanao indices but not the UniFrac and MC Visayas indices (Supplementary Table S2).

**Figure 3.**
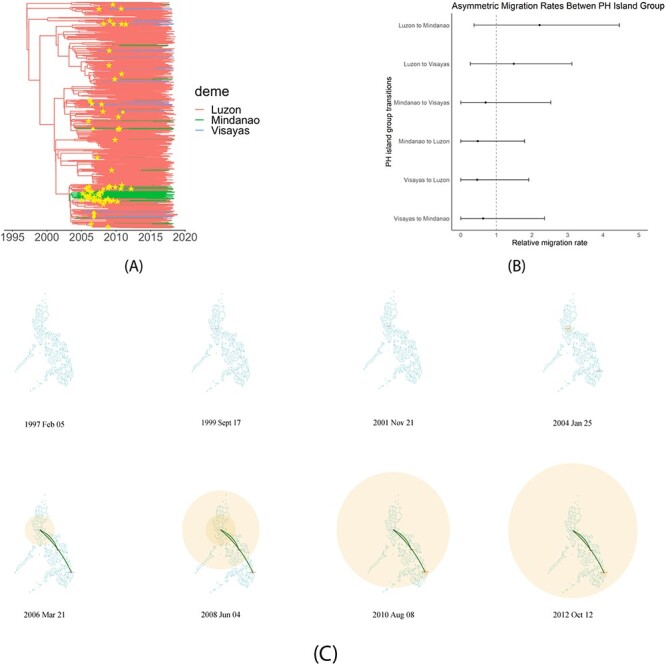
Analysis of geographic spread and relative migration rates of HIV CRF01_AE across Philippine island groups. (A) MCC tree of the largest Philippine CRF01_AE cluster from the full dataset of 1042 sequences with sample collection date and location metadata generated with BEAST under the ‘phylogeo’ model and summarized with TreeAnnotator. All nodes achieved a posterior probability support value of 1.0 except for the nodes labeled with a star symbol, corresponding to a support value greater than or equal to 0.95 and less than 1.0, and a circle symbol, corresponding to a support value greater than 0.66 and less than 0.95. The branch color is set according to the most likely geographic location of a branch at the level of Philippine island groups: Luzon, Visayas, Mindanao. (B) The forest plot with median and 95 per cent HPD estimate of the asymmetric relative migration rates of CRF01_AE between all pairs of Philippine island groups. A dashed vertical line indicates a relative migration rate equal to 1.0, or no greater or less than the mean of migration rates. (C) The phylogeographic spread of CRF01_AE between Luzon, Visayas, and Mindanao island groups over time visualized with SPREAD4. Edges represent transitions between island groups. The size of the polygons over island groups corresponds to the intensity of localized virus transmission at the specified location and time.

Phylogeographic analysis was performed with the full dataset of sequences from the large Philippine CRF01_AE cluster, excluding sequences that did not have island group metadata (*n *= 1042). The state at the root of the resulting CRF01_AE MCC tree was inferred to be the Luzon island group (posterior probability = 1.0) (Fig. 3A; Supplementary Table S1), implicating Luzon as the epicenter of the large CRF01_AE cluster. With wide overlapping 95 per cent HPD intervals, differences among the relative migration rates between three different island groups were not statistically significant (Fig. 3B; Supplementary Table S1). However, the median relative migration rates of Luzon-to-Visayas 1.3351 [0.2701, 3.1224] and Luzon-to-Mindanao 2.0072 [0.3712, 4.4567] were higher than the average rate of 1.0 and higher than the median migration rates of Mindanao-to-Luzon, Mindanao-to-Visayas, Visayas-to-Luzon, and Visayas-to-Mindanao, which were all below 1.0 (Fig. 3B; Supplementary Table S1), suggesting Luzon to have been the main exporter. The reconstructed phylogeographic spread of CRF01_AE over time shows that from the late 1990s to about 2001 the largest CRF01_AE cluster started to spread in a localized manner in Luzon (Fig. 3C). From 2001 to 2004, there was an increase in localized spread in Luzon but also the start of migration of CRF01_AE from Luzon to Mindanao (Fig. 3C). From 2004 to 2006, the localized spread in Luzon continued to increase, while both Visayas and Mindanao had already been seeded with CRF01_AE from Luzon (Fig. 3C). From 2006 onwards, there is a continued rise in the local transmission of CRF01_AE in all three island groups (Fig. 3C). To assess the robustness of these phylogeographic inferences, sensitivity analysis was performed on ten subsamples of 102 sequences with an equal number of randomly selected sequences from each island group. The root state of Luzon and the migrations of Luzon-to-Visayas and Luzon-to-Mindanao observed in the full dataset were supported in the majority of subsamples in the sensitivity analysis (Supplementary Table S1; Supplementary Fig. S3).

### Comparable trends of CRF01_AE and Subtype B *N*_e_ and *R*_e_ over time

**Figure 4.**
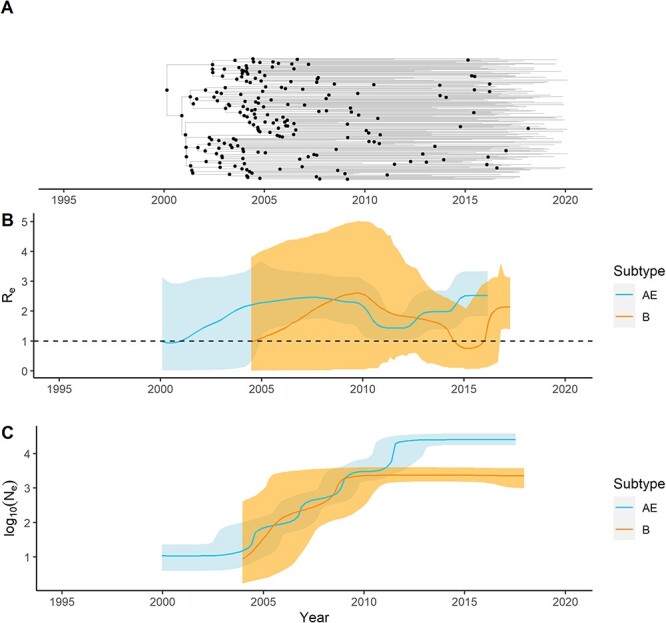
Comparison of phylodynamics between Philippine CRF01_AE and Subtype B. (A) Time-scaled MCC tree from BDSKY analysis of the full set of 192 Subtype B PR/RT sequences summarized with TreeAnnotator. Common ancestor nodes are highlighted as dots. (B) The change in the Subtype B effective reproductive number (*R*_e_) over time obtained from BDSKY analysis, superimposed over that of CRF01_AE, with the medians represented as solid lines and the 95 per cent HPDs as shaded intervals. A dashed line indicates the value of *R*_e_ equal to 1. (C) The change in Subtype B effective population size (*N*_e_) over time in log scale obtained from BSP analysis, superimposed over that of CRF01_AE, with the medians represented as solid lines and the 95 per cent HPDs as shaded intervals.

To contextualize the transmission dynamics of CRF01_AE, phylodynamic analysis was also performed on Subtype B sequences available in the RITM Molecular Biology Laboratory database, focusing on the largest monophyletic Philippine clade of Subtype B sequences (Supplementary Fig. S1). A majority of the available Subtype B sequences sampled in the Philippines (197/316, 62.54 per cent) belonged to one major clade (Supplementary Fig. S1; SH-aLRT, UFBoot branch support: 97.7/96). A second minor clade was also present (28/316, 8.89 per cent), while the rest were small clusters or singletons (91/316, 28.57 per cent). The resulting BSP analysis revealed a growth in Subtype B *N*_e_ from its tMRCA in May 1999 [May 1993, October 2003] until about 2010, throughout which it was comparable to the increasing CRF01_AE *N*_e_ (Fig. 4C). From 2010 to 2018, a plateau in Subtype B *N*_e_ is observed (Fig. 4C). The median tMRCA of Subtype B is later, and the median evolutionary rate of 2.5436E − 3 [95 per cent HPD: 1.9472E − 3, 3.1441E − 3] substitutions/site/year for Subtype B is lower but not found to be significantly different than that of CRF01_AE (Supplementary Table S1).

Phylodynamic analysis with BDSKY showed that the trend of the Subtype B *R****_e_*** also fluctuated over time in a similar pattern compared with the CRF01_AE *R*_e_ (Fig. 4B). The *R*_e_ of Subtype B increased from 1.0 around the time of its tMRCA to about 2.61 [95 per cent HPD: 0.050, 5.03] in 2009 (Fig. 4B). It then decreased to as low as 0.75 [95 per cent HPD: 0.058, 2.089] in 2015 and rebounded to 2.14 [95 per cent HPD: 1.41, 3.14] in 2016 (Fig. 4B). Although there are differences in the precise dating of shifts in dynamics, the Subtype B cluster shows the same general trend of an increasing, decreasing, and rebounding *R*_e_ as in the CRF01_AE cluster.

## Discussion

The single large monophyletic CRF01_AE Philippine clade observed in this study supports a single past introductory event that led to the majority of infections in the country. This is consistent with the pattern observed in HIV epidemics outside Africa where a few subtypes or CRFs come to predominate (Ferdinandy et al. 2015). Based on the phylogeny of sampled sequences, there is no evidence of much ongoing transmission from other sporadic introductions. The monophyly and estimated tMRCA of CRF01_AE in the mid-to-late 1990s are consistent with results from a study using nearly full-length genomes from the Philippines (Chen et al. 2019) and a recent reconstruction of CRF01_AE transmission from Africa to Asia (Junqueira et al. 2020). This is about a decade later than its expansion in Southeast Asia coming from Africa (Junqueira et al. 2020) and the earliest identified CRF01_AE sample in the country around the 1980s (Salvaña et al. 2017). The estimated evolutionary rate for CRF01_AE at about 0.003 substitutions/site/year is close to the estimate in previous studies when either the full coding region or the *gag* gene is used but not when the *pol* gene or near complete genome sequence is used (Junqueira et al. 2020; Patiño-Galindo and González-Candelas 2017). This could be due to different selected regions in the sequence or different within-host replication or epidemiological dynamics in the sampled populations (Scholle et al. 2013). Our estimate for the CRF01_AE tMRCA overlaps with that of Subtype B, and it is possible that the two large clusters began spreading at effectively the same time.

In our analysis, the confidence intervals of the *R*_e_ of the largest monophyletic CRF01_AE and B clusters overlap. The Subtype B intervals are particularly wider, likely due to a smaller sample size. The median lines of both *R*_e_ estimates are also comparable. From these results, we are unable to distinguish possible differences in the *R*_e_ of the two large clusters. More precise estimates are needed to resolve any such differences, especially if the magnitudes are not large. Use of larger sample sizes and whole-genome sequences may lead to more precise estimates of these epidemiological parameters (Dudas and Bedford 2019) as well as allow for more accurate classification of subtypes and recombinant forms (Topcu et al. 2022). More sampled sequences from Subtype B in particular will help in a more precise comparison. A recent study in 2022 by Salvaña et al. (2022) has shown evidence of significantly higher viral load in CRF01_AE infections compared to Subtype B and other circulating subtypes, which suggests potentially higher transmissibility of CRF01_AE infections. The same study by Salvaña et al. (2022) also suggested higher rates of TDR among CRF01_AE infections compared to other HIV subtypes.

The same general trend of a rise, decrease, and rebound in CRF01_AE *R*_e_ was also observed in the largest Subtype B cluster *R*_e_. This supports the notion of a genuine dip in this period, since the trend was observed in both the largest CRF01_AE cluster (in the full dataset and two distinct subsamples) and the largest Subtype B cluster independently. This decrease overlapped with the 4th and 5th Philippine AIDS Medium Term Plans developed by the Philippine National AIDS Council for 2005–10 (Philippine National AIDS Council (PNAC) 2005) and 2011–16 (Philippine National AIDS Council (PNAC) 2011), respectively. These public health interventions have had a measurable effect on transmission based on modeling by the Philippine Epidemiology Bureau, potentially having averted as much as 152,300 infections as of 2020 (Epidemiology Bureau DoH 2020a) and this may be reflected in the fluctuations in *R*_e_ that were not subtype specific. The rebound in *R*_e_ requires further investigation as influences on *R*_e_ can be complex. Some factors that could be investigated for having influenced the increase in *R*_e_ of both CRF01_AE and Subtype B after 2010 include the shift in the majority of infections to a younger age demographic (Farr and Wilson 2010; Ross et al. 2013, 2015; Epidemiology Bureau DoH 2022) and the steep rise in the proportion of MSM compared to male-to-female mode of transmission (Epidemiology Bureau DoH 2020b). The rebound suggests an intensification of factors contributing to transmission or that the effects of interventions were not sustained.

There is also an apparent lag of 2–4 years in the fluctuation of Subtype B *R*_e_ compared to CRF01_AE, but we suspect that this is an artifact of uncertainty in tMRCA estimates and that both epidemics may have been synchronically fluctuating over time. Despite this fluctuation, the CRF01_AE *R*_e_ remained above a value of 1.0, consistent with no decreasing trend observed in its *N*_e_. The Subtype B *R*_e_ crossed the value of 1.0, but this estimate has wide uncertainty intervals, and more precise estimates with a larger dataset and longer sequences would be useful in confirming this result. The growth in CRF01_AE *N*_e_ from the tMRCA to 2014 is consistent with the continuous increase in HIV-1 incidence throughout this period (Gangcuangco and Eustaquio 2023). However, the plateaus observed in both CRF01_AE and Subtype B *N*_e_ from 2014 to 2017 and from 2010 to 2018, respectively, must be interpreted with caution. BSP *N*_e_ estimates in the more recent past may be biased toward flattening due to factors such as the paucity of coalescent events at the tail end of the plot (De Silva, Ferguson, and Fraser 2012). As such, we have truncated the BSP plots for both CRF01_AE and Subtype B at their last respective coalescent events. Although both plateaus begin when there are still coalescent events on their corresponding MCC trees, the smaller uniform CRF01_AE subsamples show plateaus that begin slightly earlier compared to the full dataset. This suggests that the fewer number of coalescent events in the recent past depending on the density of sampled sequences could be involved in the plateaus. Sampling of epidemiologically linked sequences from the same localized outbreaks may also be another potential source of bias that cannot be ruled out from this analysis (De Silva, Ferguson, and Fraser 2012). Finally, the incidence of HIV-1 in the country is also known to have increased all throughout the investigated period (Gangcuangco and Eustaquio 2023). Use of more densely sampled sequences, sequences with more recent collection dates, and accounting for epidemiologically linked samples in subsequent BSP analyses may further resolve the pattern of *N*_e_ in these more recent periods. It must be mentioned that the drug resistance mutation genotyping at RITM was not performed from 2012 to 2013 due to lack of reagents, and neither were there CRF01_AE sequences from this period sourced from referring hospitals and social hygiene clinics. However, publicly available Philippine sequences from this period were available from Genbank and included in this analysis, so there was no gap in sampling from the first to the latest sample collection dates.

It is plausible as our phylogeographic analysis suggests for Luzon to have been the origin and main exporter of the CRF01_AE epidemic to Visayas and Mindanao. Many of the most dense and urbanized cities in the Philippines are found in Luzon (Philippine Statistics Authority 2022), and the busiest and most highly connected airport in the country is located here (Dona et al. 2009). Filipinos from various provinces regularly migrate to big cities such as in Metro Manila for work (UNESCO 2018). The spread of the largest CRF01_AE cluster appears to have been concentrated in Luzon from the tMRCA in the mid-to-late 1990s up to about 2001. As infections in Luzon grew and as *R*_e_ continued to increase from 2004 to 2006, CRF01_AE was seeded to Visayas and Mindanao whereupon localized onward transmission was established as well. Higher median relative migration rates for Luzon-to-Visayas and Luzon-to-Mindanao agree with this pattern of geographic spread, although the rates have wide uncertainty intervals overlapping with each other and the value of 1.0. Thus, it is still possible that the migration rates are not significantly different from each other. The wide intervals could be due to the limitations in the phylogenetic information in the partial *pol* sequences analyzed and in sampling from Visayas and Mindanao. The sensitivity analysis also supports this pattern of dissemination from Luzon, but the Mindanao-to-Visayas migration observed in 8/10 subsamples and the Visayas-to-Mindanao migration observed in 1/10 subsamples could be artifacts arising from subsampling, especially given they were not observed in the full dataset. Phylogeographic analysis with a larger number of sequences from Visayas and Mindanao and use of whole-genome sequences may help to confirm the observed pattern in geographic spread and reduce the uncertainty surrounding migration rates. Increasing sampling across 17 different Philippine administrative regions will also make the resolution of regional-level patterns of migration possible. If Luzon is confirmed as the main exporter as our results suggest, focused interventions in Luzon during this timeframe would have helped control further spread in other island groups. If further analysis, on the other hand, finds comparable migration rates across island groups or regions, this would suggest extensive mixing between locations and the need for geographically wide coverage of control measures to suppress the overall CRF01_AE epidemic in the Philippines.

To summarize, we showed that the introduction of CRF01_AE into the Philippines was in the mid-to-late 1990s, the majority of CRF01_AE sequences belong to a single large cluster, both CRF01_AE and Subtype B *N*_e_ showed an increasing growth trajectory from their tMRCA onwards, both CRF01_AE and Subtype B shared the same trend of an increasing, decreasing, and rebounding *R*_e_, and that the epicenter of the CRF01_AE epidemic was Luzon which was likely the main exporter to Visayas and Mindanao. The shift from Subtype B to the more aggressive CRF01_AE, with its faster progression to advanced immunosuppression (Salvaña et al. 2022) and its implications on ARV treatment regimens (Salvana et al. 2020), highlights the need to be vigilant on changing phylodynamics of HIV subtypes in the Philippines. The results of our study characterize the CRF01_AE-predominant epidemic, providing context for the effectiveness of past and existing public health measures and may inform future measures toward more effective control of the HIV epidemic in the Philippines.

## Supplementary Material

vead073_Supp

## Data Availability

The BEAST XML files used in this study can be found at https://github.com/mblbdmu/CRF01_AE-PH. The data underlying this article are available in the GenBank Nucleotide Database at https://www.ncbi.nlm.nih.gov/nucleotide and can be accessed with OQ428277 - OQ428541 and OQ710464- OQ711602.
