# Supplementary material for: Reconstructing the phylodynamic history and geographic spread of the CRF01_AE-predominant HIV-1 epidemic in the Philippines from PR/RT sequences sampled from 2008 to 2018
Source: Virus Evol. 2023 Dec 7;9(2):vead073. doi: 10.1093/ve/vead073 (PMC10735293; doi:10.1093/ve/vead073)
Supplement: vead073_Supp [file vead073_supp.zip › suppl_data/[VIRUS EVOLUTION] Supplementary Tables.docx]

# **Supplementary Tables**

## **Table S1.** Posterior evolutionary and epidemiological parameter estimates from all BEAST and BEAST2 analyses performed in the study, including the median and 95% HPD of each parameter under each analysis. In the phylogeography sensitivity analysis, the root state with the highest probability was Luzon in 10/10 subsamples. The median migration rates for Luzon-to-Mindanao and Luzon-to-Visayas were greater than the average rate of 1.0 for 6/10 and 9/10 subsamples, and greater than the other 4 migration rates for 9/10 and 9/10 subsamples, respectively. These same inferences were also retained in the phylogeographic analysis of one spatiotemporally uniform subsample of 255 sequences.

|  | | | **tMRCA & BSP (*N_e_*)**  **/ Coalescent Bayesian Skyline / BEAST v2.6.7** | | | **BDSKY (*R_e_*) Analysis / Birth-Death Skyline Serial**  **/ BEAST v2.6.7** | | | | | | **Phylogeography / Coalescent Bayesian Skyline**  **/ BEAST v1.10.4** | | | | |
| --- | --- | --- | --- | --- | --- | --- | --- | --- | --- | --- | --- | --- | --- | --- | --- | --- |
| Dataset (description) | Number of sequences | Date range of sequences in sample | clock rate (ucld) | clock rate std dev (ucld) | TMRCA mean (95% HPD) | clock rate (ucld) | clock rate std dev (ucld) | TMRCA mean (95% HPD) | origin of the epidemic | become uninfectious rate | sampling proportion  first; second; third sampling interval | clock rate (ucld) | clock rate std dev (ucld) | TMRCA mean (95% HPD) | Deme probability at root  [Luzon, Visayas, Mindanao] | Relative migration rates  L-M; L-V; M-V; M-L; V-L; V-M |
| CRF01_AE full dataset  (All CRF01_AE sequences with sample collection dates) | 1144 | 2008-01-15, 2018-11-18 | 3.2664E-3 [2.9275E-3, 3.5935E-3] | 0.3215 [0.282, 0.3579] | 1996.4306 [1991.9633, 1999.7731] | 3.22E-3 [2.9192E-3, 3.5245E-3] | 0.32 [0.2817, 0.3577] | 1997.7681 [1995.7821, 1999.8734] | 1996.4229 [1995.0149, 1998.5807] | 0.6046 [0.3659, 0.9642] | 0; 3.7514E-3 [1.2658E-3, 6.9174E-3]; 1.4185E-3 [4.1236E-4, 2.9262E-3] | n/a | n/a | n/a | n/a | n/a |
| CRF01_AE full dataset  (All CRF01_AE sequences with sample collection dates & island group metadata) | 1042 | 2008-05-06, 2018-11-18 | n/a | n/a | n/a | n/a | n/a | n/a | n/a | n/a | n/a | 3.7226E-3 [3.371E-3, 4.1118E-3] | 1.2853E-3 [1.0806E-3, 1.4975E-3] | 1997.306 [1993.3983, 2000.5466] | [1.0, 0.0, 0.0] | 2.0072 [0.3712, 4.4567]; 1.3351 [0.2701, 3.1224]; 0.3615 [1.5209E-4, 2.5282]; 0.2452 [6.1998E-6, 1.7949]; 0.1931 [2.1344E-5, 1.906]; 0.2919 [2.4266E-4, 2.352] |
| CRF01_AE first spatiotemporally uniform subsample  (One sequence sampled per stratum of time and island group) | 256 | 2008-01-15, 2018-11-18 | 2.9141E-3 [2.466E-3, 3.3596E-3] | 0.2143 [0.1325, 0.2895] | 1998.1608 [1994.1681, 2001.2728] | 2.7156E-3 [2.3273E-3, 3.0975E-3] | 0.2259 [0.1473, 0.2954] | 1997.0695 [1994.0385, 1999.7235] | 1994.316 [1992.5436, 1998.1053] | 0.3837 [0.1704, 0.7474] | 0; 2.0119E-3 [1.7438E-4, 5.632E-3], 1.5433E-3 [5.3528E-5, 6.4558E-3] | n/a | n/a | n/a | n/a | n/a |
|  | 255  (Excluding earliest sequence from 2008-01-15 since lacks location metadata) | 2008-05-14, 2018-11-18 | n/a | n/a | n/a | n/a | n/a | n/a | n/a | n/a | n/a | 3.3701E-3 [2.8218E-3, 3.9325E-3] | 8.0482E-4 [5.107E-4, 1.1323E-3] | 1999.3308 [1995.8073, 2002.1207] | [1.0, 0.0, 0.0] | 1.7172 [0.2787, 3.9271]; 1.5832 [0.3197, 3.6744]; 0.3378 [5.0017E-5, 2.2124]; 0.2237 [1.4684E-5, 1.9528]; 0.213 [9.4787E-7, 1.7977]; 0.3418 [3.2842E-5, 2.4005] |
| CRF01_AE second spatiotemporally uniform subsample  (One sequence sampled per stratum of time and island group, excluding sequences in the first subsample) | 151 | 2008-01-15, 2018-11-18 | 2.5345E-3 [1.9887E-3, 3.112E-3] | 0.3122 [0.2285, 0.3958] | 1998.5354 [1993.0151, 2002.729] | 2.3903E-3 [1.9004E-3, 2.8573E-3] | 0.3329 [0.2502, 0.4227] | 1998.007 [1993.8745, 2001.5995] | 1995.1656 [1983.3223, 1998.4345] | 0.3051 [0.1062, 0.5993] | 0; 1.6961E-3 [1.2902E-4, 5.571E-3]; 1.3194E-3 [1.5142E-5, 7.2946E-3] | n/a | n/a | n/a | n/a | n/a |
| Rep01  (34 randomly sampled sequences per island group) | 102 | 2008-10-02, 2018-08-29 | n/a | n/a | n/a | n/a | n/a | n/a | n/a | n/a | n/a | 2.8025E-3 [2.169E-3, 3.4747E-3] | 7.2978E-4 [3.2674E-4, 1.1737E-3] | 2001.6261 [1997.3391, 2004.8559] | [0.9986, 0.0001, 0.0013] | 1.5445 [0.2758, 3.6948]; 1.7382 [0.3052, 4.0356]; 0.3664 [9.5426E-5, 1.8458]; 0.2975 [7.4592E-5, 2.2719]; 0.1737 [1.7952E-5, 1.6545]; 0.3583 [5.3358E-5, 2.4303] |
| Rep02  (34 randomly sampled sequences per island group) | 102 | 2008-08-21, 2018-08-29 | n/a | n/a | n/a | n/a | n/a | n/a | n/a | n/a | n/a | 1.9625E-3 [7.6888E-4, 3.5538E-3] | 5.3391E-4 [1.639E-4, 1.1206E-3] | 1992.2973 [1970.3585, 2005.1] | [0.7597, 0.0779, 0.1624] | 1.2429 [2.0494E-4, 3.2594]; 0.8074 [3.6824E-7, 2.4806]; 0.6496 [6.955E-4, 2.2511]; 0.5552 [1.8592E-5, 2.7074]; 0.844 [0.0179, 2.4407]; 0.5207 [1.8054E-4, 2.5484] |
| Rep03  (34 randomly sampled sequences per island group) | 102 | 2008-09-23, 2018-06-18 | n/a | n/a | n/a | n/a | n/a | n/a | n/a | n/a | n/a | 3.0173E-3 [2.2854E-3, 3.825E-3] | 5.5668E-4 [1.8687E-5, 9.8877E-4] | 2002.9124 [1997.7335, 2006.5174] | [0.9761, 0.0063, 0.0176] | 1.3133 [0.0389, 3.1745]; 1.7697 [0.229, 4.228]; 0.3894 [2.2519E-5, 1.9462]; 0.3595 [2.9601E-5, 2.3622]; 0.2481 [4.9501E-6, 1.3883]; 0.446 [3.1631E-5, 2.5979] |
| Rep04  (34 randomly sampled sequences per island group) | 102 | 2008-11-20, 2018-06-26 | n/a | n/a | n/a | n/a | n/a | n/a | n/a | n/a | n/a | 2.5431E-3 [1.7558E-3, 3.2965E-3] | 6.5797E-4 [3.1674E-4, 1.0821E-3] | 2000.0259 [1991.4562, 2005.3953] | [0.4992, 0.3695, 0.1313] | 0.6992 [9.2629E-5, 2.3736]; 1.2274 [1.704E-5, 3.9366]; 0.6065 [6.4615E-5, 2.307]; 0.4246 [1.123E-4, 2.2809]; 0.8794 [1.4024E-4, 3.5817]; 0.4967 [4.8012E-5, 2.4708] |
| Rep05  (34 randomly sampled sequences per island group) | 102 | 2008-05-14, 2018-06-18 | n/a | n/a | n/a | n/a | n/a | n/a | n/a | n/a | n/a | 2.5601E-3 [1.6848E-3, 3.4206E-3] | 5.2852E-4 [1.5207E-4, 9.8491E-4] | 1999.2383 [1992.0463, 2003.9569] | [0.9096, 0.0019, 0.0885] | 0.8045 [3.947E-4, 2.3896]; 2.1532 [0.3631, 4.9426]; 0.4911 [8.2544E-5, 2.4185]; 0.4157 [7.293E-4, 1.8611]; 0.3188 [2.3965E-5, 2.3938]; 0.3186 [1.8579E-4, 1.8096] |
| Rep06  (34 randomly sampled sequences per island group) | 102 | 2013-03-06, 2018-05-15 | n/a | n/a | n/a | n/a | n/a | n/a | n/a | n/a | n/a | 4.0385E-3 [2.7546E-3, 5.3964E-3] | 8.6722E-4 [2.7319E-4, 1.5578E-3] | 2006.7683 [2002.8331, 2009.5646] | [0.9608, 0.0051, 0.0341] | 0.9588 [0.049, 2.568]; 1.7328 [0.1943, 4.2352]; 0.8189 [2.9502E-5, 2.538]; 0.4532 [7.9368E-5, 2.4036]; 0.2334 [4.3773E-6, 1.7475]; 0.3871 [4.7698E-5, 2.4229] |
| Rep07  (34 randomly sampled sequences per island group) | 102 | 2008-09-23, 2018-05-23 | n/a | n/a | n/a | n/a | n/a | n/a | n/a | n/a | n/a | 2.8478E-3 [2.21E-3, 3.5738E-3] | 4.0644E-4 [2.763E-8, 7.6789E-4] | 2001.7421 [1997.8764, 2004.8435] | [0.9504, 0.0002, 0.0493] | 1.1536 [0.0704, 2.8785]; 1.4254 [0.1666, 3.4488]; 1.005 [0.0181, 2.6603]; 0.5272 [1.6632E-4, 2.3306]; 0.1949 [7.7753E-6, 1.5331]; 0.3464 [7.7513E-5, 2.4774] |
| Rep08  (34 randomly sampled sequences per island group) | 102 | 2008-09-23, 2018-05-15 | n/a | n/a | n/a | n/a | n/a | n/a | n/a | n/a | n/a | 3.0652E-3 [2.0819E-3, 4.0533E-3] | 5.3645E-4 [1.4815E-5, 9.6586E-4] | 2001.9963 [1995.9312, 2006.0278] | [0.9743, 0.0018, 0.0239] | 1.2018 [0.0856, 3.1084]; 1.8694 [0.298, 4.472]; 0.4507 [6.314E-5, 2.2044]; 0.3608 [2.0999E-5, 2.1707]; 0.2156 [2.3308E-5, 1.8068]; 0.3433 [8.6637E-5, 2.3799] |
| Rep09  (34 randomly sampled sequences per island group) | 102 | 2009-07-21, 2018-05-18 | n/a | n/a | n/a | n/a | n/a | n/a | n/a | n/a | n/a | 3.2714E-3 [2.4491E-3, 4.1147E-3] | 6.6471E-4 [5.4088E-5, 1.1212E-3] | 2004.1384 [1999.6734, 2007.3051] | [0.7909, 0.0921, 0.117] | 0.9279 [7.0191E-4, 2.9461]; 1.6685 [7.6139E-5, 3.9727]; 0.5104 [1.0373E-4, 2.2238]; 0.657 [1.016E-5, 2.5664]; 0.2937 [7.8306E-6, 2.5707]; 0.3441 [1.3202E-5, 2.1382] |
| Rep10  (34 randomly sampled sequences per island group) | 102 | 2009-07-21, 2018-06-18 | n/a | n/a | n/a | n/a | n/a | n/a | n/a | n/a | n/a | 3.3147E-3 [2.4887E-3, 4.2189E-3] | 6.9015E-4 [1.3072E-4, 1.1805E-3] | 2003.0291 [1998.8634, 2006.2572] | [0.8793, 0.0119, 0.1088] | 1.7707 [0.017, 4.1576]; 1.0867 [8.8777E-4, 2.8649]; 0.5606 [6.7666E-5, 2.3193]; 0.3984 [8.5057E-6, 2.6313]; 0.2499 [3.2371E-5, 1.3625]; 0.4651 [1.6107E-5, 2.7313] |
| Subtype B full dataset  (All subtype B sequences with sample collection dates) | 192 | 2008-07-29, 2020-02-03 | 2.5436E-3 [1.9472E-3, 3.1441E-3] | 0.3362 [0.2487, 0.4192] | 1999.3638 [1993.3917, 2003.7948] | 2.6171E-3 [2.0119E-3, 3.2113E-3] | 0.3653 [0.2777, 0.4572] | 2000.5173 [1995.0924, 2004.1765] | 1993.6913 [1989.7996, 1996.689] | 0.4589 [0.215, 0.7931] | 0; 4.6176E-3 [6.8062E-4, 0.0118]; 0.0111 [2.2509E-3, 0.0271] | n/a | n/a | n/a | n/a | n/a |

## **Table S2.** Befi-BaTS analysis using island group as trait, 1000 trees downsampled from the BSP posterior set of trees of the full set of 1144 CRF01_AE sequences and the uniform subsample of 256 sequences, and 999 simulated null trees. Test statistics include association index (AI), Slatkin-Maddison parsimony score (PS), unique fraction (UniFrac), nearest taxa (NT), net relatedness (NR), phylogenetic diversity (PD), and maximum monophyletic clade size (MC) indices.

| Statistic | observed.mean | lower.95..CI | upper.95..CI | null.mean | lower.95..CI.1 | upper.95..CI.1 | significance |
| --- | --- | --- | --- | --- | --- | --- | --- |
| Full dataset of 1144 CRF01_AE sequences | | | | | | | |
| AI | 32.47795104980469 | 30.275352478027344 | 34.6763801574707 | 42.27787780761719 | 40.324859619140625 | 44.201812744140625 | **<0.001** |
| PS | 282.0118408203125 | 272.0 | 292.0 | 304.89459228515625 | 297.9892272949219 | 311.4252014160156 | **<0.001** |
| UniFrac | 0.6108807921409607 | 0.5916100144386292 | 0.6289796829223633 | 0.5153625011444092 | 0.48905497789382935 | 0.540738582611084 | 1.0 |
| NT (combined, distance-based) | 3613.3154296875 | 3338.29931640625 | 3914.974365234375 | 3680.8193359375 | 3646.185791015625 | 3714.92333984375 | **<0.001** |
| NR (combined, distance-based) | 3509393.5 | 3120044.5 | 3958466.25 | 3480122.25 | 3466126.0 | 3494217.5 | 0.5455455780029297 |
| PD (combined) | 9525.5029296875 | 8925.14453125 | 10180.19140625 | 9745.76953125 | 9701.220703125 | 9791.82421875 | **<0.001** |
| MC (Luzon) | 37.324005126953125 | 36.0 | 40.0 | 16.956676483154297 | 14.050592422485352 | 21.11733055114746 | **0.0020020008087158203** |
| MC (Visayas) | 3.041980743408203 | 2.0 | 4.0 | 1.268737554550171 | 1.0118407011032104 | 1.9956942796707153 | **0.0010010004043579102** |
| MC (Mindanao) | 3.902045249938965 | 3.0 | 5.0 | 1.866779088973999 | 1.4004305601119995 | 2.1765339374542236 | **0.0010010004043579102** |
| Uniform subsample of 256 CRF01_AE sequences | | | | | | | |
| AI | 8.541756629943848 | 7.251990795135498 | 9.784134864807129 | 13.465739250183105 | 12.297884941101074 | 14.61048698425293 | **<0.001** |
| PS | 76.56842803955078 | 70.0 | 84.0 | 101.57260131835938 | 97.15684509277344 | 105.66333770751953 | **<0.001** |
| UniFrac | 0.5604402422904968 | 0.5175217986106873 | 0.602721095085144 | 0.3664216995239258 | 0.3099447786808014 | 0.42195451259613037 | 1.0 |
| NT (combined, distance-based) | 969.5881958007812 | 855.9962768554688 | 1095.1585693359375 | 987.002685546875 | 971.0637817382812 | 1003.9395751953125 | **0.007007002830505371** |
| NR (combined, distance-based) | 129519.71875 | 113407.1171875 | 131727.78125 | 131727.78125 | 130514.921875 | 132945.140625 | **<0.001** |
| PD (combined) | 2493.047119140625 | 2240.0771484375 | 2773.701904296875 | 2576.713134765625 | 2555.3720703125 | 2597.944091796875 | **<0.001** |
| MC (Luzon) | 13.733266830444336 | 9.0 | 19.0 | 8.290800094604492 | 6.5144853591918945 | 10.920080184936523 | **0.017017006874084473** |
| MC (Visayas) | 2.1598401069641113 | 2.0 | 3.0 | 1.639060139656067 | 1.152847170829773 | 2.103896141052246 | 0.2112112045288086 |
| MC (Mindanao) | 5.658341884613037 | 4.0 | 8.0 | 2.2350621223449707 | 1.8461538553237915 | 2.9510488510131836 | **0.0010010004043579102** |
