# Supplementary figures and images for: Reconstructing the phylodynamic history and geographic spread of the CRF01_AE-predominant HIV-1 epidemic in the Philippines from PR/RT sequences sampled from 2008 to 2018
Source: Virus Evol. 2023 Dec 7;9(2):vead073. doi: 10.1093/ve/vead073 (PMC10735293; doi:10.1093/ve/vead073)

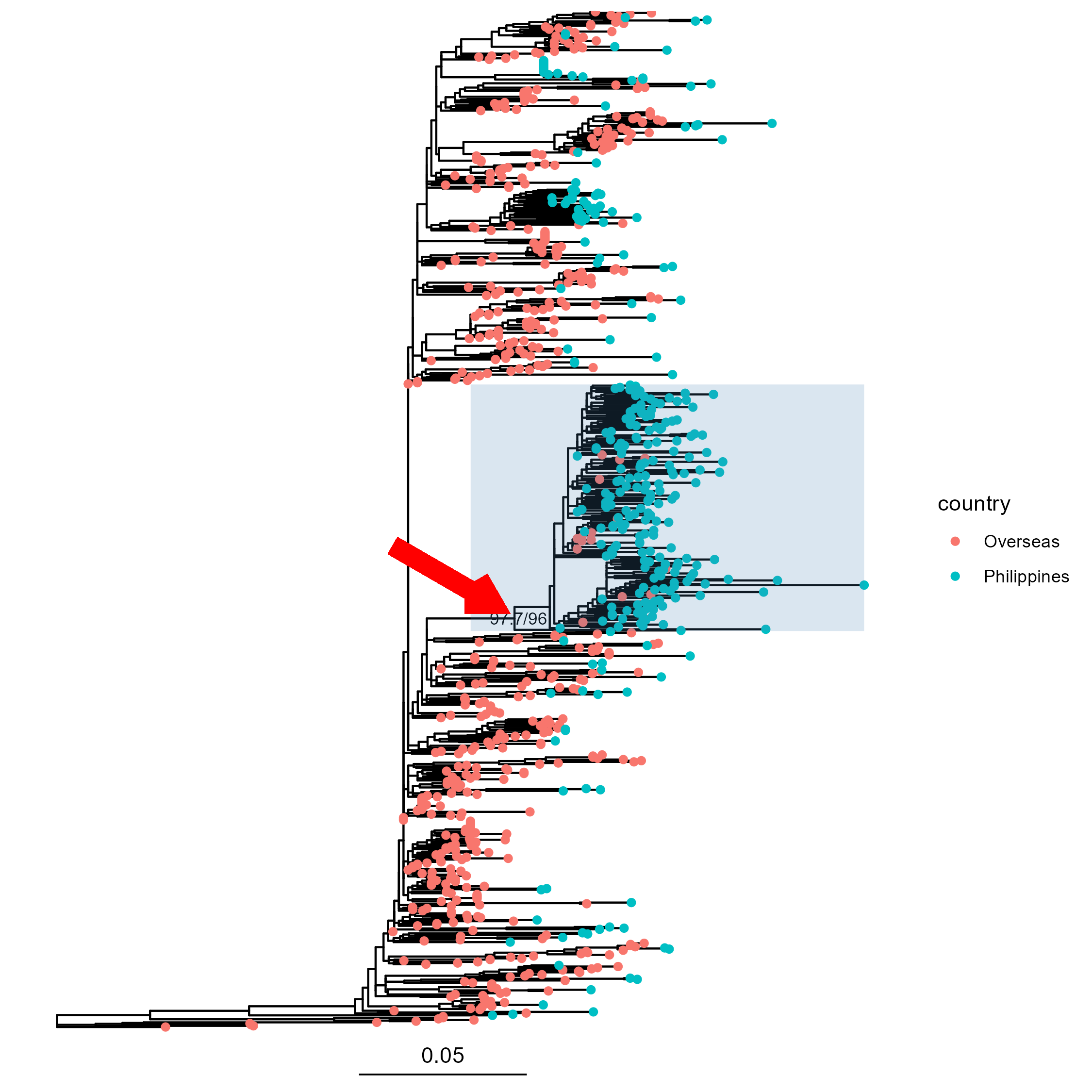

Supplement: vead073_Supp [file vead073_supp.zip › suppl_data/figS1.png]

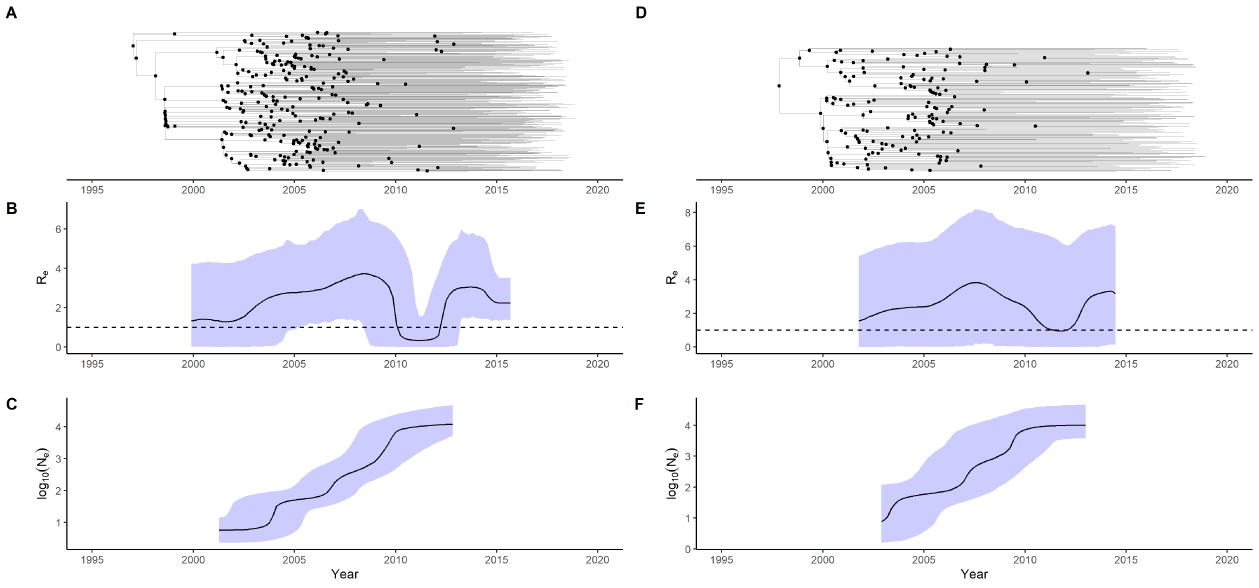

Supplement: vead073_Supp [file vead073_supp.zip › suppl_data/figs2300.png]

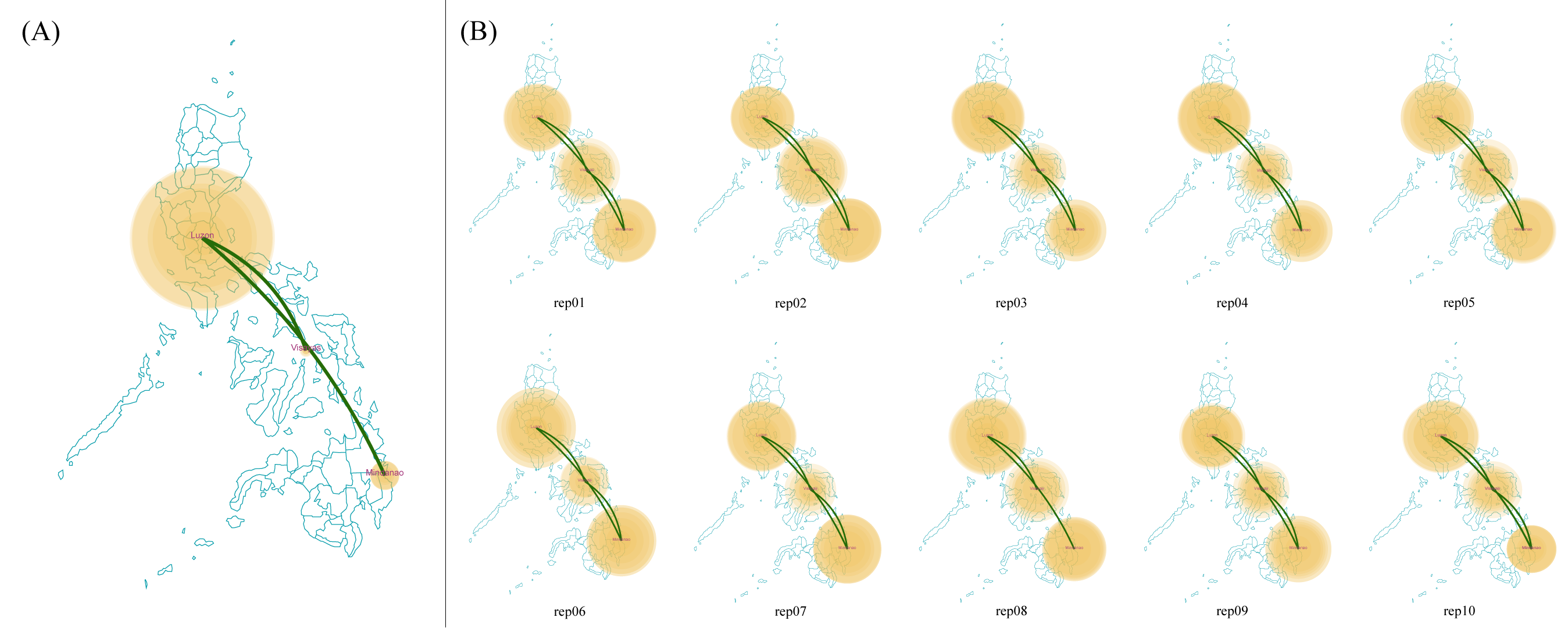

Supplement: vead073_Supp [file vead073_supp.zip › suppl_data/figs3300.png]
